# Organoids – the future of pre-clinical development of AAV gene therapy for CNS disorders

**DOI:** 10.1038/s41434-025-00527-8

**Published:** 2025-03-27

**Authors:** Vivienne M. Kaiser, Anai Gonzalez-Cordero

**Affiliations:** 1 Stem Cell Medicine Unit, Children’s Medical Research Institute, Westmead, NSW Australia; 2 Faculty of Medicine and Health, The University of Sydney, Sydney, NSW Australia

**Keywords:** Neurological disorders, Neural stem cells, Gene therapy

## Abstract

Advancements in our understanding of genetic disease and adeno-associated virus has prompted great excitement into the field of AAV-mediated gene therapy, particularly for genetic diseases of the central nervous system, including retinal disorders. Despite significant progress, exemplified by the approval of therapies such as Luxturna® and Zolgensma®, a substantial number of therapies remain in pre-clinical or early clinical stages, with many failing to advance to later phases. Whilst the use of animal models to test safety and delivery route efficacy of AAV treatments is imperative, differences in tissue structure and physiology between humans and animal models has restricted precise disease modelling and gene therapy development for many CNS disorders. Alongside the FDA push for non-animal alternative models, researchers are increasingly turning to human-based models, including stem cell-derived organoids, which can offer a more accurate representation of human cellular microenvironments and niches. As such, this review explores the advantages and limitations of brain and retinal organoids as pre-clinical models of disease, with a primary focus on their utility in identifying novel AAV capsids, cell-specific promoters, and their role in recent pre-clinical AAV gene therapy studies.

## Introduction

Owing to our comprehensive understanding of genetic disease and viral systems, adeno-associated virus (AAV)-mediated gene therapy has become a reliable avenue for treatment of previously incurable genetic diseases. Numerous pre-clinical studies and the FDA-approval of six AAV gene therapies (Luxturna®, Zolgensma®, Hemgenix®, Elevidys®, Beqvez® and Adstiladrin®) have generated significant excitement into the development of novel gene therapies, particularly for disorders of the central nervous system (CNS), which have been at the forefront of gene therapy innovation.

The CNS is comprised of the brain, spinal cord and retina, where it serves a significant role in detecting stimuli, emotion, memory, communication and thought processing. CNS disorders have one of the highest rates of morbidity and mortality globally, resulting in over 11.1 million deaths in 2021 [1]. In fact, reducing premature mortality from these types of non-communicable diseases by 2030 has become a sustainable development goal of the United Nations. Inherited retinal disorders (IRDs) affect approximately 1 in 2000 individuals [2], with an expected 5.5 million people suffering worldwide [3]. Neurological disorders are genetically heterogeneous, with IRDs alone having over 260 causative genes. Consequently, research into gene therapies for these disorders has significantly accelerated, with many using AAV vectors, which are currently the gold-standard for gene therapy treatment, due to their relatively high safety profile, broad tissue tropism and strong transgene expression without chromosomal integration [4]. Additionally, the CNS has become an attractive target organ for AAV, as it is relatively immune-privileged. Target cells, such as neurons in the brain and photoreceptor cells in the retina, are also post-mitotic in adulthood leading to strong transgene expression. The retina, in particular, is also an accessible and contained organ for subretinal or intravitreal delivery. Despite these attractive properties, it is becoming increasingly evident that many gene therapy trials fall short at the pre-clinical stage, as is evidenced by the many studies not progressing to later-stage clinical trials. Whilst reasons for this include the inability of many therapeutics to evade neutralising antibodies or to cross blood neural barriers, a concern is the lack of pre-clinical models that accurately recapitulate the human state.

Previously, rodents and larger animal models, including non-human primates (NHPs) have been the first-choice models for pre-clinical development. Whilst the use of these models is imperative to test safety and route of administration, differences in tissue structure and physiology between humans and animal models has restricted correct disease modelling and gene therapy/vector development. For example, in the retina, many studies have found that rodents lack calyceal processes– microvilli that surrounds the base of photoreceptor outer segments that are implicated in many retinal ciliopathies, including Usher Syndrome, a deaf-blinding disease accounting for 50% of all deaf-blinding cases [5]. As a result of an undetectable mouse retinal phenotype, there have been great difficulties in understanding disease mechanisms and developing therapies for this disease. Additionally, many studies looking at AAV transduction efficiency have also shown differences between species [6–8]. Encouragingly, the FDA Modernisation Act (FDAMA) 2.0, enacted in 2022, amended previous requisites for therapies to be tested in animals prior to use in humans. Very recently, the US Senate passed the FDAMA 3.0 further building upon the previous legislation aimed to establish a clear process for approving non-animal testing methods, thus, resulting in an increase in momentum of using alternate, non-animal methods for pre-clinical discovery. As such, many scientists are exploring the use of human-based pre-clinical models to develop and test gene therapies, including 2D cell cultures, ‘humanised’ organs in animal models and stem-cell derived organoids.

In this review, we evaluate the use of CNS organoid models for the pre-clinical development of AAV gene therapies, making note of their physiological and functional relevance to human tissues, and how advancements in the engineering of organoids are rapidly progressing, leading to more robust models. We will then assess their utility in the identification of novel capsids, cell-specific promoters and, more importantly, we investigate their role in testing novel AAV gene therapies for a range of genetic CNS disorders. Finally, we explore the current challenges of these models and discuss how overcoming the technological limitations will expand the possibilities for disease modelling and AAV pre-clinical development.

## Human PSC-derived CNS Organoid Models

The brain and retina are complex and sensitive organs with intricate cellular and molecular environments, therefore replicating their dynamic interactions and functions outside of the body has proven difficult. Whilst neural 2D cell culture is relatively cheap and reproducible, and thus has been valuable for high throughput use in drug screening platforms, it lacks tissue complexity and organisation thus impacting cell morphology, survival, proliferation and differentiation [9]. As such, protocols for the differentiation of 3D brain and retinal organoids have progressed rapidly. Organoids, albeit only replicating early stages of organogenesis, are sophisticated, self-assembling human organ models with structural and physiological niches, similar to in vivo tissues and organs. They can be derived from embryonic or induced pluripotent stem cells (PSCs); the latter of which is of particular interest for gene therapy purposes as they can be derived from affected individuals with disease-causing-mutations [10]. In addition, many organoids have proven to be functional, with brain organoids demonstrating network connectivity through bursts of electrical activities in multi-electrode arrays (MEAs) [11–13] and photoreceptor cells in retinal organoids showing response to light comparable to in vivo tissue when patch clumping is performed [14]. However, current organoids are far from being perfect models of organ complexity as cellular connectivity with other organs or systems is missing. In light of this, many groups are attempting to bridge this gap through the creation of ‘assembloids’, the engineering of complex organoids, and multicellular models grown on a chip.

### Brain organoids

**Fig. 1:**
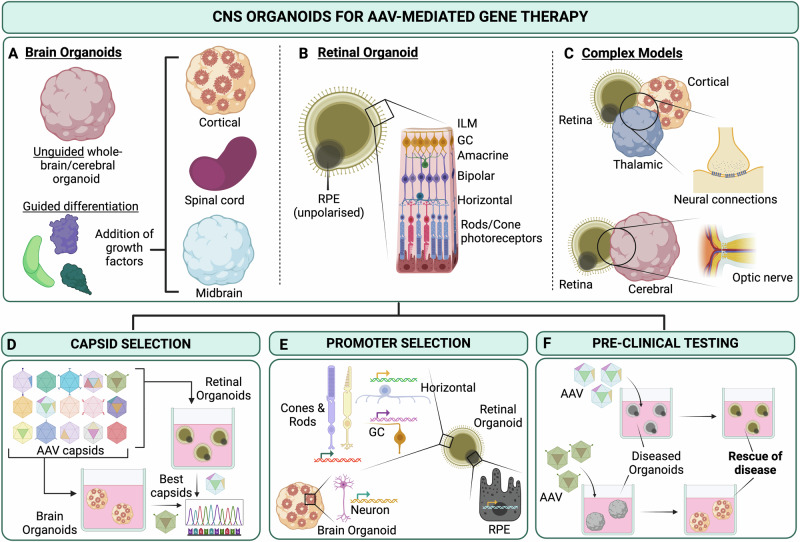
Uses of CNS organoids in AAV-mediated gene therapy. **A** Brain organoid differentiation follows unguided or guided protocols. **B** Retinal organoids contain seven cell layers with lamination. **C** Complex models comprised of neural organoids to achieve different neural connections between organs. **D** Different AAV capsid modifications tested on retinal and neural organoids to identify most specific and selective capsid. **E** Identification of cell-specific promoters for retinal and neural organoids. **F** AAV-mediated gene therapy on retinal and neural organoids to restore disease phenotype. Created with Biorender.com.

Neural organoid differentiation follows unguided or guided protocols (Fig. 1A). Unguided organoid differentiations depend on the spontaneous self-organisation of the PSC-aggregates and thus contain heterogeneous brain region identities [15]. The first unguided protocol, and the first 3D whole cerebral organoid, was developed by Lancaster et al., using embryoid bodies from iPSCs [16, 17]. These organoids contained regions reminiscent of forebrain, midbrain, hindbrain, choroid plexus, and even retina. Many studies have since sought to optimise protocols to enhance cellular diversity and functionality. Quadrato et al., displayed neural progenitor cells, astrocytes, oligodendrocyte-like precursors, excitatory neurons and photosensitive retinal cells [12]. Ormel et al., developed cerebral organoids containing microglia [18] and Sharf et al. generated organoids with mature dendritic spines and inhibitory/excitatory synapses [11]. Through MEAs and calcium imaging, the functionality, including spontaneous network activity of these organoids was confirmed [11–13]. Whilst these organoids offer an overview of the whole brain and are useful to test AAV capsid tropism and promoter specificity in various brain regions, such approach can be hindered by organoid-to-organoid variability requiring careful quality controls.

Guided differentiation protocols have been developed to create region-specific organoids. For example, Sloan et al., used SMAD inhibition followed by a SHH agonist to generate either dorsal (cortical) or ventral (subpallial) forebrain organoids [19]. Here, it was shown that these organoids contained astrocytes and recapitulated the interactions of glutamatergic and GABAergic neurons observed in vivo. Currently, there are numerous other studies that have generated differentiation protocols for distinct brain regions, including the forebrain, midbrain, spinal cord and the retina [13, 19–26]. These protocols are reliant on expensive growth factors; however, precise control leads to lesser organoid and batch-to-batch variability which is advantageous for disease modelling and to test efficacy of therapies. Therefore, the choice of which organoids to use is an essential step towards developing an appropriate gene therapy.

### Retinal organoids

Similar to guided neural organoid differentiation protocols, retinal organoids can be generated using a number of protocols, including self-forming retina structures obtained from guided 3D cultures or unguided 2D/3D environments. These protocols can all sufficiently recapitulate the human retina, comprising all seven cell types and retinal lamination (Fig. 1B), and their differentiation has been reviewed extensively [27–29]. Additionally, these organoids show physiological responses to light stimuli [30, 31] and retinal ganglion cells (RGCs) have been shown to exhibit electrophysiological responses reminiscent of those in normal retinal neurons as examined by whole-cell recordings [32]. More recently, many groups have sought to improve retinal organoid models to enhance modelling capabilities. Through antioxidant and lipid supplementation, West et al., developed retinal organoids with improved outer segments, thus enabling modelling of IRDs, including X-linked *retinitis pigmentosa GTPase regulator* (*RPGR)* [33]. Moreover, there is strong push to develop retinal organoids with higher reproducibility - a factor that is particularly important for the wider uptake and regulatory approval of these models. Indeed, through quick reaggregation methods and controlled BMP4 signalling, Harkin et al., developed a protocol that resulted in retinal organoids of 100% purity with highly consistent size, shape and cellular composition [34]. Despite these advancements, there is still work to be done to generate retinal organoids that contain retinal pigment epithelial (RPE) cells, which will allow for the recapitulation of the polarity and orientation of the human retina. In current models, RPE cells predominately form clumps in the periphery of organoids (Fig. 1B) instead of enveloping the retina and photoreceptor cells. Whilst the lack of RPE cells enables easy access of viral vectors to the photoreceptor cells located in the outer most layer of the organoid, this model fails to mimic the constraints of subretinal injection in the eye - the primary method to deliver AAVs in diseases affecting both RPE and photoreceptor cells. Additionally, retinal organoids do not have a macula, a region of high visual acuity only found in primates, thus precluding extensive research into genetic diseases affecting the macular region, such as inherited Stargardt disease, a juvenile maculopathy leading to central vision loss arising from RPE and cone photoreceptor degeneration in the macula.

### Complex models

Whilst protocols for CNS-specific regions are progressing rapidly, there is the limitation that these organoids do not model the intricate interplay between the organoid and neighbouring systems and tissues. This is particularly crucial as many CNS disorders affect multiple regions of the brain. To navigate this, many groups have created complex models of multi-organoid systems, or ‘assembloids’, which connect various CNS organoid regions together to study long distance communications and thus enhance modelling capabilities (Fig. 1C) [35–41]. For example, in retinal organoids, RGCs are lost as cultures mature, most likely due to the lack of interaction with output regions in the brain. As such, Fligor et al., fused retinal, cortical and thalamic organoids, where it was observed that RGCs had enhanced survival, were responsive to environmental cues and were able to generate axon projections into the neural assembloids [39]. Moreover, following an adherent 2D-3D protocol, Fernando et al., demonstrated the spontaneous formation of retinal and brain organoids forming in proximity in culture. Once matured in 3D, the RGCs in the centre of the retinal organoid were found to send axonal projections into the brain organoid [40]. Many groups have also used assembloids to more accurately recapitulate disease. For example, Wu et al., developed striato-nigral assembloids that recapitulated Huntington’s Disease spiny neuronal projection defects, previously not seen in striatal organoids alone [41]. Taken together, these studies highlight that assembloids can provide a more comprehensive and integrated model for studying complex brain functions and diseases affecting multiple brain regions. These multi-organoid systems will highlight AAV transduction preferences when different organs are exposed concomitantly, something that to date has not been tested in vitro. Additionally, through the combination of organoids and microfluidics, organ-on-a-chip models are being used to increase the longevity of in vitro tissues through more efficient nutrient diffusion and to recapitulate the dynamic fluid flow seen in in vivo organs. As such, modelling the blood brain (BBB) and retinal barriers (BRB) has become possible, which is particularly important as these tissue-blood interfaces have proven to be a major hurdle in identifying brain and retina specific AAV capsids for systemic administration.

## Organoids for AAV gene therapy

Arguably one of the most valuable utilities of human PSC-derived organoids is their use in pre-clinical development of novel gene therapies. Thus, many researchers are using organoids for capsid selection, identification of cell-specific promoters and to test efficacy of novel gene therapies.

### Selection of novel capsids

One significant consideration when designing gene therapies, is the serotype of the AAV particle. Within the AAV genome, the *Cap* gene encodes three overlapping structural proteins: VP1, VP2 and VP3 [42]. These proteins are responsible for assembly of the 60-mer icosahedral shell and are arranged in the ratio 1:1:10, respectively. All AAV capsids have a core beta-barrel motif, with strands connected by variable surface loops which produce capsid surface topological variations between serotypes. These variations are implicated in receptor binding and antibody recognition, and hence define serotype specificity. Most serotypes use proteoglycan receptors (HSPG) or O/N-linked sugars as their primary receptor and growth factors, integrins and/or laminin receptor as a co-receptor [43]. Indeed, there are currently 13 characterised native serotypes which are able to target different cell types through expression of their respective AAV receptors [44]. It is well known that there are species differences in the glycosylation patterns of these cell receptors [45], yet our predominant understanding of AAV tropism has come from animals. As such, it is unsurprising why capsid variants identified in in vivo animal models, do not always show tropism in other models, or even strains. For example, Stanton et al., found that well-performing capsids, such as AAV.CAP-B10 and AAV.CAP-B22, identified in mice models and shown to have tropism in the marmoset CNS, were not able to transduce the cynomolgus macaque CNS [7]. Additionally, the expression of the GPI-anchored Ly6a protein in the vasculature of permissive mice strains leads to efficient AAV-PHB and AAV-PHB.eB transduction across the BBB, whereas no transduction of these capsids is observed in mammalian models, including NHPs [8]. Therefore, using a more relevant model, such as human organoids, to classify AAV tropism is needed to understand the mechanisms behind human AAV transduction.

#### Organoids to test and screen for novel AAV capsids

Since our general understanding of AAV tropism has predominantly come from animal models, human organoids, despite their foetal-like phenotypes, are becoming increasingly accepted as pertinent models for the screening of engineered AAV capsids and transduction efficiency (Fig. 1D) [6, 46, 47]. Our group tested transduction efficiencies in vitro in a number of mouse and human retinal cells of six commonly used in vivo AAV capsids [48]. In mouse and human RPE cells and retinal organoids, ShH10 was the most efficient capsid, transducing most retinal cells in the organoids including photoreceptor cells. Both AAV2/8 and the tyrosine mutated AAV2/8 T were poor performers in vitro which differs from its widespread tropism in the murine retina and its common use in subretinal injections to treat both photoreceptor and RPE cells. Another study compared AAV5 and AAV9 capsids – currently used in clinical trials – and found higher levels of AAV5 vector DNA, transgenic mRNA and protein expression in cerebral organoids [49]. This finding was consistent in two different transgenes and with both cytomegalovirus (CMV) and hybrid CMV enhancer/chicken β-actin (CAG) constitutive promoters. Again, this is contrary to the widespread use of AAV9 in vivo to treat CNS diseases. Stanton et al., identified AAV6 to have the highest transduction efficiency in neurons in cortical organoids, despite the documented neural tropism of AAV2 [46, 50]. Interestingly, using isogenic and Alzheimer’s disease cortical organoid models, they also showed that AAV6 transduction efficiency was impacted in this disease state; a finding that may lead to the future development of disease-specific capsids.

Organoids are also being used to identify capsids in relation to the expression of their respective glycan receptors. Indeed, Garita-Hernandez et al., found that AAV2(7m8) efficiently transduced retinal organoids and that this coincided with an increase in expression of sydnecan-3 and laminin receptor 1 [51]. This study also examined AAV9 efficacy, a previously used capsid to deliver genetic material to cone photoreceptor cells. Interestingly, they observed that the AAV9 N-linked galactose primary receptor is only found in mature cones, hence only 226-day old organoids were efficiently transduced by AAV9. CNS organoids are also proving to be valuable in identifying novel, engineered capsids with higher efficiency. For example, Völkner et al., tested different peptide modifications of AAV9 in retinal organoids and identified AAV9.NN to have the highest efficiency and fastest onset kinetics with detectable transgene expression two days post-transduction in photoreceptors, interneurons and Müller glia cells [52]. Another group used spinal cord organoids and found that four novel capsids (AAV9.NL1, AAV9.TH1, AAV9.TP1 and AAV9.TR2) were able to transduce motor neurons and glia at a higher efficiency than AAV9 [53].

Recently, Westhaus et al. and Drouyer et al., created three ‘AAV Testing Kits’ containing non-specific and CNS and retina-specific kits, each containing 51, 13 and 25 wild-type, natural and bioengineered capsids, respectively [6, 47, 54]. GFP-tagged barcoded capsids enabled high-throughput next-generation sequencing of the samples to identify vector performance based on cellular entry (DNA) and transgene expression (RNA). Westhaus et al., identified that AAV2(7m8) transduced retinal organoids, murine retina and primary and iPSC-derived RPE cells with the most efficiency at both cellular entry and transgene levels, thus supporting previous data that this is the most efficient retinal capsid [51, 55]. Unexpectedly, however, transduction of AAV2(7m8) was not observed in the retinal organoid cone photoreceptors. Importantly, this study also identified other novel AAV candidates, AAV2-L2 and AAV2-M4, that showed selective tropism towards human cells over mouse cells at a high efficiency comparable to AAV2(7m8).

To navigate the caveat that many AAVs struggle to cross the inner limiting membrane (ILM) of the retina when delivered intravitreally, the authors added the kit capsids to the vitreal side of polarised human retinal explants. Once again, results showed AAV2(7m8) to be the strongest transducer. However, this method may not be the best indicator of ILM-crossing due to the potential concern that the AAVs could have leaked and transduced the underside of the explant in the transwell.

Using cortical and cerebral organoids, Drouyer et al., performed an AAV screen using all three testing kits [6]. Interestingly, the top performing capsid in these organoids was also AAV2(7m8), followed by AAV2-retro. This finding highlights the promiscuous nature of AAV2(7m8) at least in vitro, as this result did not translate to mouse and NHP in vivo studies whereby novel capsids AAV2.M1 and AAV.SYD12 were the top performing capsids. This is likely owing to the differences in complexity, size and administration route of in vivo models. As such, further research needs to be conducted to elucidate the working mechanisms of each capsid to understand their specific tropisms.

Whilst these AAV testing kits have been instrumental in identifying novel AAV candidates, there is the caveat that these studies only provided a snapshot of AAV transduction after a few weeks in culture. Expression kinetics is an important factor to understand peak levels of transduction and to determine the appropriate time of gene therapy analysis in vitro. As such, Rogler et al., developed a 3D quantification system to assess AAV transduction efficiency in live retinal organoids at single-cell resolution using confocal microscopy and machine learning, tracking it spatially and temporally over 40 days [56]. They tested three highly efficient capsids, AAV2(7m8), AAV9.NN and AAV2.NN, and found that the modified AAV2.NN outperformed the other serotypes with 82% transduction efficiency and in onset time (seven days). Deep learning technologies combined with live-imaging will offer a robust way of measuring capsid tropism however comparisons with quantitative technologies such as flow cytometry will be needed to validate this process.

Taken together, these studies highlight that neural tissues and brain organoids appear to be receptive to AAV transduction in vitro with most capsids tested performing relatively well. Contrary, retinal organoids are more difficult to transduce and capsids which are commonly used in vivo in rodents are not very efficient in vitro. For transduction of retinal organoids, the use of AAV2(7m8) is clearly recommended to maximise efficiency of gene therapy treatments. Table 1 provides a comprehensive summary of the various capsids that have been tested in vitro in CNS organoids and in vivo.

**Table 1:** Efficient AAV Capsids for transducing human CNS organoids and other animal models.

Retinal Gene Therapy						
*Study*	Human Organoid		Mouse in vivo			
	*Capsid*	*Titre (vg/org)*	*Capsid*	*Titre (vg/eye)*		
Lane et al. [66]	AAV2/5	1E11				
Kruczek et al., [67]	AAV2	1E11				
Rodrigues et al., [71]	AAV2(7m8)	4E9				
West et al., [33]	AAV2(7m8)	3E11				
Boon et al., [70]	AAV5	3.3E10				
Sladen et al., [68]	AAV2(7m8)	3E11				
Sai et al., [69]	AAV2(7m8)	1E11				
Duan et al., [72]	AAV2(7m8)	5E9				
Tornabene et al., [78]	AAV2/2	1E12	AAV2/8	4.3E9		
Reidmayr et al., (2023)	AAV9	1.5E12 (1 AAV)	AAV8(Y733F)	6E10 (1 AAV)		
		3E12 (2 AAVs)		6E10 (2 AAVs)		
Ivanchenko et al., (preprint)	AAV9.PHB.B	8.5E11 (1 AAV)				
		1.7E12 (2 AAVs)				

Neural Gene Therapy						
Latour et al., [73]	AAV9	3E10				
Depla et al., [49]	AAV5	6E11				

Libraries						
Retinal						
*Study*	*Human Organoid*	*Mouse intravitreal*	*Mouse subretinal*	*Human Retinal Explant*	*iPSC RPE*	*Primary RPE*
Westhaus et al., [47]	AAV2, AAV4, AAV2(7m8), AAV-HRS1, AAV-KP1, AAV-KP3, AAV13, AAV2-L1, AAV2-M4	AAV2, AAV5, AAV2(7m8), AAV2-M1	AAV5, AAV8, AAV2, AAV-shH10, AAV2-M1, AAV2-L4, AAV2-L5	AAV2, AAV2(7m8), AAV-CD-SYD09, AAV6, AAV2-L1, AAV2-M4, AAV2-L2	AAV1, AAV5, AAV2, AAV2(7m8), AAV-CD-SYD09, AAV6/AAV-CD15; AAV2-L2, AAV2-M4, AAV2-1.3	AAV2, AAV-HRS1, AAV2(7m8), AAV-KP1, AAV-KP3, AAV2-M4, AAV2-1.3
Gonzalez-Cordero et al., [48]	AAV2/8, shH10, AAV2/2		AAV2/8			
McClements et al., [64]	AAV2(7m8)					
Garita-Hernadez et al., [63]	AAV2(7m8), AAV9 (mature cone photoreceptors only)					
Völkner et al., 2021	AAV9.NN					
Rogler et al. (preprint)	AAV2.NN					

Brain						
*Study*	*Cerebral Organoid*	*Cortical Organoid*	*Spinal Cord*	*Mouse*	*NHP*	
Drouyer et al. [6]	AAV-DJ, AAV-LK03, AAV2, AAV2-retro, AAV-Anc80L65; AAV2(7m8), AAV-SYD04, AAV-SYD11, AAV-SYD12, AAV-NP59, AAV2-L1, AAV2-M4, AAV2-L5, AAV2-M1	AAV-DJ, AAV-LK03, AAV2, AAV2-retro; AAV2(7m8), AAV2-L1, AAV2-M4, AAV2-L5, AAV2-M1		AAV2-M1	AAV-CD-SYD09, AAV2-N582S, AAV2-7m8, AAV2-M1, AAV-SYD12	
Depla et al., [49]	AAV5					
Stanton et al., (preprint)		AAV6				
Hanlon et al., [53]			AAV9.NL1, AAV9.TH1, AAV9.TP1, AAV9.TR2			

### Promoter selection

Alongside cell- and species-specific capsids, promoters are also extremely important drivers of efficacious gene therapies. There are many different kinds of promoters, including constitutive or inducible and ubiquitous or cell specific. The majority of current gene therapies in clinical trials utilise constitutive, ubiquitous promoters, including CMV, CAG and chicken β-actin (CBA). Reasons for use of these promoters reside in the fact that they show strong transgene expression using a lower dose – a seemingly attractive factor for clinical translatability. However, they can lead to off-target transduction and deleterious effects in certain disease states. In particular, the CNS contains many genes that are dose-sensitive and highly regulated. Indeed, it has been shown that mild exogenous overexpression of MECP2, a protein implicated in Rett Syndrome, in in vivo models can have deleterious effects, which is presenting a critical issue in the development of gene therapies for this disease [57, 58]. In addition to this, some promoters, including CMV, may not be active under certain cellular conditions or ages. For example, our group found that GFP driven by the CMV promoter was only able to achieve transgenic expression in mature PSC-derived photoreceptor cells [59], which may suggest why Westhaus et al., did not see transgene expression in organoid cone photoreceptors by AAV2(7m8) [47]. Further to this, there are many concerns of methylation of CMV promoters over time, which can silence the transgene [60]. Therefore, it is critical that either endogenous or cell-specific promoters are used as they enable the expression of physiological levels of transgene, thus respecting the biology and necessities of the cells.

Previously, transduction of CNS neurons has been achieved using the human synapsin (*hSYN*) promoter [61] whereas the rhodopsin kinase (*GRK1*) promoter has been used to target photoreceptor cells specifically in retinal organoids [62]. The use of these promoters has been fundamental in transgene efficacy; however, most genes have undescribed promoters, and it is inherently difficult to identify specific promoter regions, therefore studies are now using organoids to test cell-specific promoters to ensure cell-selectivity and sensitivity in a human tissue context.

#### Organoids to identify cell-specific promoters

Since organoids contain most cell-types of the organ in vivo, they are becoming increasingly popular for the identification of cell-specific promoters (Fig. 1E). Garita-Hernandez, et al., showed that PR1.7 cone-specific promoter, was able to drive high-level specific expression of GFP in red and green (L/M opsin)-cone photoreceptors, which enabled cell sorting to enrich this population for cell transplantation [63]. Additionally, EFS, CAG and *GRK1* promoters were tested in retinal organoids, where it was observed that CAG-driven transgene delivered via AAV2(7m8), AAV2 quad mutant, AAV5 and AAV8(Y733F) was present in a broad range of cell-types, whereas *GRK1* was specific to photoreceptor cells [64], thus confirming previously identified results [59, 62]. Whilst EFS is also considered a constitutive, ubiquitous promoter, reporter transgene expression was minimal in the retinal organoids, which is interesting considering the strong in vivo transgene expression observed from this promoter [65]. It can also be noted that organoids can be useful for confirming previously identified cell-specific promoters and establishing a suitable transduction timeline. For example, Gonzalez-Cordero et al., demonstrated that GFP driven by PR2.1.opsin which was previously described to transduce only L/M Opsin cones, was also able to transduce S Opsin cones. hRhodopsin promoter was also shown to mediate robust expression in retinal organoid rod photoreceptors [59]. They also found that cones can be transduced at early timepoints, whilst rod transduction was only observed at later timepoints. Whilst research into different promoter regions using retinal organoids is well underway, curiously, the same progress has not been seen in brain organoids, perhaps owing to the current successes of neuronal-specific promoters in vivo. Despite this, current neuron-specific promoters, such as *hSYN*, are pan-neuronal, meaning they do not distinguish between subtypes of neurons. As such, the use of region-specific brain organoids provides a useful platform for the identification of gene and cell promoters with higher specificity.

Indeed, when developing new gene therapy constructs, the choice of AAV capsid and promoter are crucial first steps. These once trivial decisions, as the landscape in pre-clinical models targeting the CNS was well known, now need to take into consideration the human cellular context. The advent of human organoids enables the robust evaluation of these two requisites in human cells offering different perspectives from animal models.

## Testing AAV gene therapy in human organoids – current landscape

As organoids are increasing in popularity, many groups are now seeking to use CNS organoid models to test AAV gene therapy efficacy.

### Traditional AAV gene augmentation

**Fig. 2:**
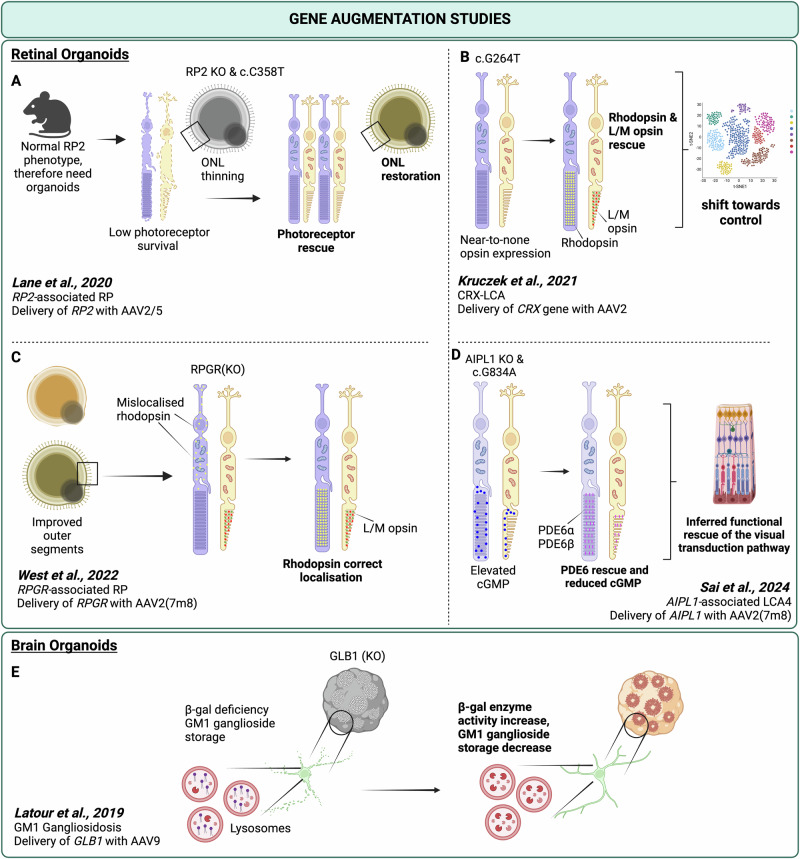
AAV-mediated gene augmentation studies using CNS organoids. **A** Delivery of RP2 gene with AAV2/5 to retinal organoids with RP2(KO) and patient (c.C358T) mutation. RP2 delivery led to increased photoreceptor survival, reduced ONL thinning and reversal of the degeneration phenotype [66]. **B** Delivery of CRX gene to CRX-LCA patient (c.G264T) displayed an increase in Rhodopsin and L/M opsin protein expression. ScRNA-seq revealed a shift of the disease organoids towards the control [67]. **C** West et al., 2022 [33] delivered AAV2(7m8) vector with RPGR to retinal organoids with improved outer segments, where correct Rhodopsin localisation was observed. **D** Sai et al., 2024 delivered *AIPL1* with AAV2(7m8) to AIPL1-associated LCA4 retinal organoid KO and patient (c.G834A) models and observed rescue of phosphodiesterase 6 alpha and beta subunits and the reduction of elevated cGMP levels, thus inferring functional rescue [69]. **E** Delivery of GLB1 using AAV9 to GLB1(KO) cerebral organoids resulted in increased of β-galactosidase enzyme activity and decreased GM1 ganglioside storage [73]. Created with Biorender.com.

Organoids can provide a relatively quick and cost-effective readout of gene augmentation efficacy. As such, many groups have used CNS disease organoid models to deliver full length healthy genes and restore disease phenotypes (Fig. 1F). The first proof-of-concept gene augmentation study using organoids was conducted in 2020, where Lane et al., delivered the *GTPase ARL3 activator* (*RP2)* to gene-edited *RP2*(KO) and patient-derived iPSC retinal organoids using AAV2/5 [66] – an interesting choice considering the low transduction efficiency observed in retinal organoids previously [48]. Despite this, the authors did observe increased photoreceptor survival and decreased ONL thinning; a novel finding owing to the absence of an *RP2* X-linked RP animal model (Fig. 2A). A year later, Kruczek et al., established an iPSC-derived retinal organoid model of *CRX*-Leber Congenital Amaurosis; a rare early-onset retinal dystrophy [67]. Owing to the congenital nature of the disease, the authors were able to demonstrate the impaired maturation of photoreceptors and lack of expression of visual opsins, consistent with animal models (Fig. 2B). The capsid choice in this study was a AAV2 as it outperformed AAV8 when driving GFP under a CMV promoter. The authors tested human *CRX* promoter sequences and formed a composite promoter to drive the human *CRX* gene in their AAV construct. Upon delivery of this construct, Rhodopsin and L/M Opsin positive cells were observed in treated organoids. It is worth nothing that they also used single cell RNA sequencing (scRNA-seq) and saw a significant shift of photoreceptor cells in the treated organoids towards the healthy control organoids. These initial proof-of-concept studies have been pivotal as they not only demonstrated the utility of organoids for modelling of early-onset diseases, but also demonstrated their ease and usefulness as potential pre-clinical tools for gene therapy.

Since, many groups have now expanded on these studies and begun pre-clinical development of gene therapies using optimised organoid protocols, more efficient AAV capsids and more robust assays to measure disease phenotype rescue. Owing to their optimised outer segments protocol, West et al., saw RPGR protein expression in the connecting cilia of photoreceptors and correction of the Rhodopsin mislocalisation phenotype upon *RPGR* delivery with AAV2(7m8) under the control of the *GRK1* promoter in *RPGR*-associated X-linked retinal organoids [33] (Fig. 2C). Using this same protocol, Sladen et al., delivered phase III clinical *RPGR* construct, Botaretigene Sparoparvovec, to *RPGR*-(KO) and isogenic control iPSC-derived retinal organoids. Unlike the trial that uses AAV5, the authors encapsulated the construct in AAV2(7m8), thus confirming the preference for this capsid due to its high efficiency in in vitro retinal organoids [68]. Recently, Sai et al., modelled *aryl hydrocarbon receptor interacting protein-like 1* (*AIPL1*) Leber congenital amaurosis subtype 4 (LC4) in retinal organoids with the aim of developing a gene therapy for this severe and early-onset degeneration. The therapeutic construct used an AAV2(7m8) capsid and a *GRK1* promoter to deliver *AIPL1*. Disease led to an accumulation of cyclic guanosine 3’, 5’-monophosphate (cGMP) levels as a result of the reduction in phototransduction PDE6 proteins in diseased organoids. cGMP is known to be elevated in many IRDs, however this was the first time that cGMP was measured in retinal organoids. Following therapy, a reduction in cGMP was observed, thus suggesting rescue of the visual functional pathway (Fig. 2D) [69]. Further development of well described functional assays will be crucial to evaluate gene therapy efficacy.

Despite the exponential popularity of using retinal organoids for gene augmentation studies, including for other IRDs such as *CRB1*-early-onset RP or LCA [70], *PRPF31*-RP [71] and *RS1* X-linked retinoschisis [72], to date, only one group have used brain organoids to test for gene augmentation, perhaps since many neurological disorders require assembloid models for accurate disease recapitulation. Latour et al., used a *GLB1*-KO cerebral organoid model, where they were able to model fatal neurodegenerative disorder, GM1 gangliosidosis, equipped with ß-galactosidase deficiency and GM1 ganglioside storage [73]. Despite not containing microglia, which is an extremely important component of modelling this disease, the authors did demonstrate increased ß-galactosidase activity and decreased GM1 ganglioside storage, upon transduction with AAV9-GLB1, consistent with trials performed in murine and feline GM1 gangliosidosis models (Fig. 2E).

Whilst organoids are proving to be incredibly useful for understanding the molecular and cellular phenotypic rescue of disease in AAV gene augmentation studies, studies to date have not taken advantage of functional readouts, such as electrophysiology. This specialised experimentation is difficult to perform and streamlined approaches, such as MEAs, are still to be adapted in organoids. For example, in retinal organoids, light response in photoreceptor cells is measured in RGCs through bipolar cells, requiring proper connectivity. Furthermore, RGCs are located in the centre of the organoids and therefore contact of these cells with electrodes requires manual dissections which can lead to disruption of the network.

### Gene knockdown

**Fig. 3:**
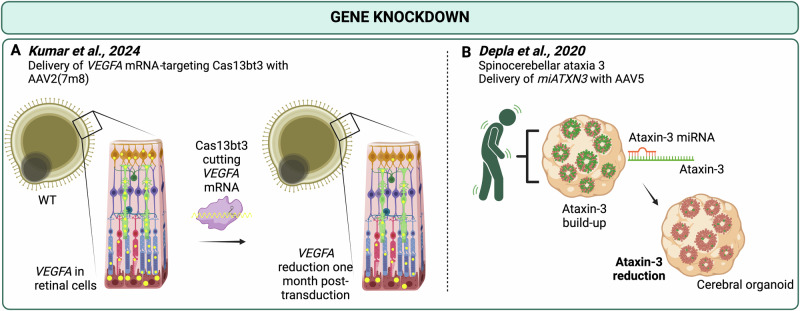
AAV-mediated gene knockdown therapy using CNS organoids. **A** Delivery of *VEGFA* mRNA-targeting Cas13bt3 with AAV2(7m8) in retinal organoids led to decreased *VEGFA* expression one-month post-transduction, particularly in Müller glia and bipolar cells [74]. **B** Delivery of a miRNA targeting Ataxin-3 using AAV5 into spinocerebellar ataxia 3 cerebral organoids with an accumulated ataxin-3 phenotype. Reduced Ataxin-3 was observed [49]. Created with Biorender.com.

Unlike gene augmentation studies, silencing or knockout of disease genes and/or RNA has not yet made the same progress in CNS organoids. This may, in fact, be due to the paucity of information regarding how certain neurological disorders manifest, the heterogeneity of disease or the regulatory issues faced with off-target effects of gene editing approaches, such as CRISPR/Cas9 proteins, in a potential clinical setting. Nevertheless, a recent study tested RNA gene editing (CRISPR/Cas13) efficiency targeting h*VEGFA* in vivo in a murine proliferative retinopathy model, and in vitro in HEK293FT cells and retinal organoids. Using AAV2(7m8) in retinal organoids, the Cas13bt3 *VEGFA* sgRNA construct efficiently reduced the expression of *VEGFA* one month post transduction, with no off-target effects observed (Fig. 3A). [74]. Further analysis using scRNA-seq on the retinal organoids showed that not only did the control non-targeting sgRNA lead to unspecific RNA binding, but that there were more DEGs relative to HEK293FT cells, particularly in macroglial and bipolar cells, thus supporting the use of organoids to obtain a more specific and sensitive readout of RNA knockdown efficiency.

Another proof-of-concept knockdown study, this time using a microRNA targeting *ATXN3*, focused on developing a treatment for spinocerebellar ataxia 3 (SCA3). Via AAV5 delivery into whole cerebral organoids, which exhibited an accumulated mutant Ataxin-3 phenotype, the authors were able to find high levels of transgene expression and a 30% reduction in Ataxin-3 expression nine days post-transduction (Fig. 3B) [49]. It can be noted, however, that SCA3 is predominantly a cerebellar, brain stem and spinal cord disease, therefore, testing of this therapy in cerebral organoids enabled limited phenotype rescue evaluations. The use of other CNS organoids or assembloid models would be required to accurately assess efficacy for this therapy. As such, the choice of which organoid to use is a fundamental one to accurately recapitulate the disease and measure phenotypic rescue.

### AAV for large genes

**Fig. 4:**
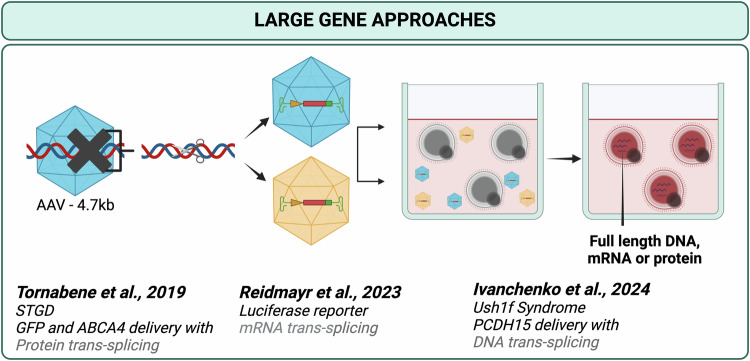
Developing novel large gene AAV-mediated gene therapy approaches using CNS organoids. Novel AAV techniques were established in retinal organoids from three groups using a dual vector approach. Ivanchenko et al., 2024 demonstrated DNA trans-splicing in Usher1f retinal organoids [76], Reidmayr et al., 2023 demonstrated mRNA trans-splicing using a luciferase reporter [79] and Tornabene et al., 2019 demonstrated protein trans-splicing with GFP and ABCA4 for Stargardt disease [78]. Created with Biorender.com.

Many genes affected in the CNS, particularly genes responsible for IRDs, are very large and often do not fit inside an AAV capsid (capacity of ~4.7 kb). As such, the delivery of dual AAV vectors has been studied in numerous animal models (reviewed in [75]). To date, many groups have used retinal organoids to test these approaches (Fig. 4). Ivanchenko et al., delivered dual hybrid DNA trans-splicing AAV9.PHB.B capsids to retinal organoids each containing half of the *PCDH15* gene (5.9 kb) – implicated in Usher1f Syndrome [76]. The authors described efficient targeting of photoreceptor cells, emergence of nascent calyceal processes and proper protein expression in these areas through immunogold scanning electron microscopy, which is a particularly exciting finding considering murine models lack calyceal processes. However, they did use a wild-type retinal organoid model, and as such, no functional rescue, or even molecular phenotypic rescue of the gene therapy could be determined. Not only that, but the results in this study are likely not representative of the DNA trans-splicing method in a disease model, as wild type gene expression may lead to the homeostatic regulation of the construct.

Many groups have recently described difficulties in efficiency of DNA trans-splicing methods for the delivery of full-length protein when using dual AAV systems [77], and as such, the use of split-inteins has become increasingly popular. Tornabene et al., was the first to test the efficiency of this approach using AAV-*GRK1*-*EGFP* intein vectors in control retinal organoids [78]. Reconstitution of EGFP fluorescence was observed in organoids and quantified via western blot. After validating the protein trans-splicing vectors, they treated Stargardt disease retinal organoids with the *ABCA4* gene (6.8 kb) using two split-intein vectors where full length protein was detected in all transduced organoids. Whilst they did not measure any phenotypic rescue in the organoids, they did see an improved retinal phenotype after subretinal delivery using AAV2/8 in in vivo STGD1 murine models, as measured by reduced RPE lipofuscin accumulation, increased thickness of the outer nuclear layer and pupil constriction. Evidently, the titres used for retinal organoids was almost 250-fold higher in comparison to in vivo, which may have posed a financial limitation hindering further experiments using the organoid model (Table 1).

Whilst split-inteins are demonstrably efficient dual AAV therapies, they do not offer much flexibility for the split site selection and can potentially generate bacterial proteins and hence, an unwanted immune response [79]. As such, Reidmayr et al., developed a dual REVeRT (reconstitution via mRNA trans-splicing) AAV system [79]. They tested this approach through the dual delivery of luciferase using AAV9 in D215 retinal organoids and noted that the REVeRT AAVs could transduce highly differentiated cells. *ABCA4* REVeRT constructs tested in a murine model of Stargardt disease showed reconstitution of full length ABCA4 and improvement of retinal degeneration. Encouragingly, they also observed rescue of function using optical coherence tomography and electroretinography, thus providing a proof-of-concept for the REVeRT method.

One major issue that has become evident in the testing of these dual AAV approaches in organoid models is the high titre of AAVs that are required in comparison to in vivo models (see Table 1 for summary). Even though organoids are marginally smaller compared to in vivo tissue, it is extremely curious as to why they require higher doses and as such, further research is warranted to investigate the mechanisms of AAV transduction in human organoids, particularly if we are to decrease the financial burden of using these models to test novel large gene approaches.

## Limitations of CNS organoid models for AAV gene therapy

### Modelling adult-onset diseases

**Fig. 5:**
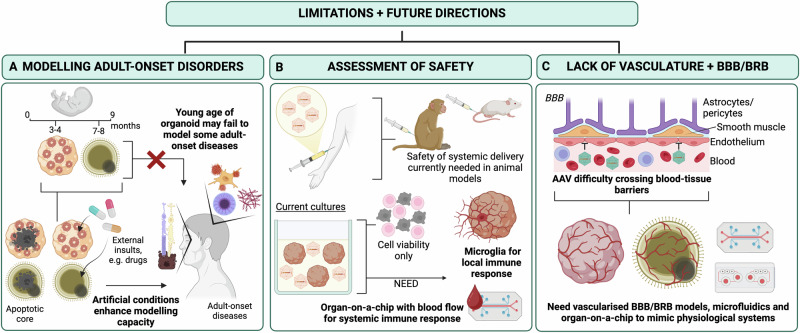
Limitations and future directions of CNS organoid models. **A** Organoid models mimic the foetal state of an organ and may fail to model some adult-onset diseases. However, artificial conditions, such as the apoptotic core and the addition of insult drugs can allow for sufficient disease modelling. **B** Animal models have a systemic immune system and hence are used for assessing the safety of AAV therapies. For organoids to bridge this gap, they need incorporation of microglia to assess local immune response and organ-on-a-chip with blood flow to assess systemic immune response. **C** Many AAV capsids struggle to cross the BBB/BRB. Organoid models are now being created that contain these interfaces and are vascularised. Microfluidic and organ-on-a-chip devices are needed to mimic these physiological systems. Created with Biorender.com.

By nature, organoids mimic the normal development of a human organ, and so, despite the remarkable developments in culture conditions, organoids still resemble foetal stages, thus limiting the modelling capacity and hence, treatment, of later onset and age-related neurodegenerative disorders, including AD and macular degeneration (Fig. 5A). Additionally, CNS organoid culture conditions are very long. For example, in the retina, cultures take up to seven to eight months to reach a stage where photoreceptor cells are sufficiently mature and contain the light-sensing outer segments. These structures are crucial for modelling many IRDs that affect the cilia and outer segments of photoreceptor cells. Moreover, brain organoids require, on average, three to four months to present functional features. As such, improving protocols that follow the natural timeline of an organ to model later-onset diseases is just not feasible. However, in vitro cultures most likely mimic a degenerative and stressed environment, as, despite culture improvements, organoids present apoptotic cores and lack crucial cell types such as blood vessels. These artificial conditions potentially mimic the disease environment in vivo contributing to the onset of disease phenotypes. Additionally, organoid models are proving to be valuable in investigating early biomarkers of many neurodegenerative diseases, most of which have typically been thought to manifest at a later stage. For example, cerebral organoids modelling sporadic and familial AD show robust expression of disease hallmarks, such as amyloid-ß plaque accumulation, tau hyperphosphorylation and neurodegeneration [80–82]. Interestingly, retinal organoids derived from familial AD patients also had these phenotypes [83]. Further, some culture protocols have also been adapted to include stressors, such as starvation and insult drugs, to accelerate the onset of late disease, including cigarette smoke extract that was used to induce macular degeneration-related damage in retinal organoids [84]. Although, despite these advancements, the genetic, epigenetic and environmental factors of ageing will preclude stem-cell derived organoids from being a ‘perfect’ model of late-onset diseases and will thus need to be paired with other animal models.

### Assessment of AAV gene therapy safety

Whilst organoids are useful for finding capsids and promoters that are most efficient at a cellular and tissue level, they currently do not provide evidence as to which capsids would perform best systemically, nor do they allow for an understanding of the immune response (Fig. 5B). Currently, assessment of AAV gene therapy safety is determined using animal models, however, this neglects the fact that there are not only species differences between immune systems, but individual differences as well. As such, some AAV gene therapies have led to immune system rejection and severe toxicity in clinical trials [85]. Current measures of AAV safety in organoids rely on cell viability assays, which is not enough to assess how a patient’s immune system would respond to the treatment. To improve these models, the incorporation of immune cells is warranted. Currently, protocols are being developed to incorporate microglia into CNS organoids – the resident immune cells of the CNS. One such study incorporated microglia into retinal organoids and showed upregulation of pro-inflammatory markers in response to endotoxin stress [86]. Additionally, many groups have generated microglia-containing brain organoids, however, many have resulted in inconsistent results as was extensively reviewed by Zhang et al., in 2023 [87]. Whilst the incorporation of microglia is important to identify a local immune response, understanding the systemic immune response to AAV gene therapies within individuals is crucial if we are to prevent toxicities associated with immune system rejection. As such, further technological advancements are warranted to create complex organoid models that contain the systemic immune system and better mimic in vivo interactions, through microfluidic and organ-on-a-chip models.

### CNS organoid models lack vasculature and blood-tissue barriers

A major hurdle in clinical translation of AAV gene therapies is the inability for many capsids to cross the BBB or BRB when administered systemically, thus posing a limitation for testing different capsids on singular or complex CNS organoids as they do not contain this blood-tissue interface (Fig. 5C). Additionally, intravitreal administration of AAV2(7m8) has also resulted in intraocular inflammation and other retinal immune responses in clinical trials due to its inability to cross the ILM in the retina [55]. To navigate this limitation, efforts are being undertaken to generate assembloids that contain a BBB/BRB or have vasculature. For example, Sun et al., incorporated microglia-like cells and endothelial cells in cerebral organoids where they not only found enhanced cell growth and increased maturity, but they were able to more accurately recapitulate phenotypic changes observed in tauopathies [88]. Additionally, Dao et al., generated BBB assembloids that contained the complete neuro-vascular unit and mimicked key characteristics of the BBB, including tight-junction complexes, pericyte processes and astrocyte end-feet ensheathing [89]. However, despite recent efforts, many vascularised assembloid protocols do not exhibit BBB-like permeability, which is an essential component for assessing AAV transduction in the brain. Microfluidics may provide a solution to this issue, as was demonstrated by Hajal et al., however, this model failed to fully capture the interactions of perfused molecules with other cell types nor did it contain the glymphatic flow seen in vivo [90]. As such, incorporating BBB/BRB microfluidics with organ-on-a-chip models, that provide precise vasculature-like perfusion and mimic the physiological response of an organ is imperative if we are to accurately model this system. Indeed, Arık et al., created an organ-on-a-chip model of the outer BRB that contained human immortalised RPE and human endothelial cells, separated by a semi-permeable membrane [91]. During oxidative stress, they reported increased barrier permeability, thus successfully mimicking hallmarks of advanced macular degeneration. Additionally, Achberger et al., created a retina-on-a-chip which not only allowed perfusion, but also improved the organoid model by recapitulating the interactions between the photoreceptors and RPE, a key characteristic of the visual cycle [92]. Using this model, the authors characterised different AAV serotypes and, consistent with previous organoid results, found AAV2(7m8) to be the most efficient [93]. Although despite this tremendous creation, this model was only able to mimic subretinal delivery. As such, further research is warranted to improve these models so that we can accurately recapitulate the physiological responses that occur when AAV is administered systemically or intravitreally.

## Conclusions and perspectives - can organoids replace current pre-clinical models?

In a time where the drive to minimise animal use for research purposes is increasing, organoid models are becoming increasingly popular tools for studying disease and testing novel therapies. Undisputedly, the development of organoid models has significantly enhanced the way researchers can identify molecular, structural, cellular and functional pathways of previously undefined CNS diseases. Their self-assembling capacity and ability to replicate the niche microenvironment of an organ has been instrumental for providing a human-relevant platform compared to traditional animal models. Arguably, they are currently the closest models to a human and as has been previously demonstrated, are valuable for the identification of cell-specific AAV capsids and promoters. Indeed, they have also shown utility for the testing of AAV therapies with phenotypic correction at a cellular level. However, organoids struggle to address some major hurdles associated with CNS AAV gene therapy, including the inability to accurately model later-onset diseases, the lack of a systemic immune response, and the absence of CNS barriers, such as the BBB and BRB/ILM of the retina. Furthermore, as highlighted by the FDAMA 3.0, there is a need for more robust non-animal alternatives that meet stringent regulatory requirements. As such, to fully integrate organoids into the gene therapy pipeline, more efforts are needed to not only improve the modelling capabilities of the organoids through microfluidics and organ-on-a-chip, but to develop standardised, reproducible protocols with quality control and potency assays. Additionally, education to researchers, clinicians and regulatory bodies is crucial in the application and interpretation of organoid models to maximise their potential impact. Despite current limitations, as these models continue to evolve, there is no doubt that their integration into the gene therapy pipeline will be of utmost importance, particularly now that animal models are no longer required for clinical translation.

## Data Availability

The data discussed in this review was obtained from the published articles as referenced.
